# Reactor Designs and Configurations for Biological and Bioelectrochemical C1 Gas Conversion: A Review

**DOI:** 10.3390/ijerph182111683

**Published:** 2021-11-07

**Authors:** Azize Ayol, Luciana Peixoto, Tugba Keskin, Haris Nalakath Abubackar

**Affiliations:** 1 Department of Environmental Engineering, Dokuz Eylul University, Izmir 35390, Turkey; azize.ayol@deu.edu.tr; 2 Centre of Biological Engineering (CEB), University of Minho, 4710-057 Braga, Portugal; luciana.peixoto@deb.uminho.pt; 3 Department of Environmental Protection Technologies, Izmir Democracy University, Izmir 35140, Turkey; tugba.keskingundogdu@idu.edu.tr; 4 Chemical Engineering Laboratory, BIOENGIN Group, Faculty of Sciences and Centre for Advanced Scientific Research (CICA), University of A Coruña, 15008 A Coruña, Spain

**Keywords:** syngas fermentation, microbial chain elongation, hydrogenotrophic methanation, bioreactors, electromethanogenesis, microbial electrosynthesis, biofilm, gas–liquid mass transfer, biocathode

## Abstract

Microbial C1 gas conversion technologies have developed into a potentially promising technology for converting waste gases (CO_2_, CO) into chemicals, fuels, and other materials. However, the mass transfer constraint of these poorly soluble substrates to microorganisms is an important challenge to maximize the efficiencies of the processes. These technologies have attracted significant scientific interest in recent years, and many reactor designs have been explored. Syngas fermentation and hydrogenotrophic methanation use molecular hydrogen as an electron donor. Furthermore, the sequestration of CO_2_ and the generation of valuable chemicals through the application of a biocathode in bioelectrochemical cells have been evaluated for their great potential to contribute to sustainability. Through a process termed microbial chain elongation, the product portfolio from C1 gas conversion may be expanded further by carefully driving microorganisms to perform acetogenesis, solventogenesis, and reverse β-oxidation. The purpose of this review is to provide an overview of the various kinds of bioreactors that are employed in these microbial C1 conversion processes.

## 1. Introduction

There is a growing trend towards research and development of waste management, as well as concurrent treatment and transformation to value-added products. Syngas, a gas mixture primarily composed of CO, H_2_, and CO_2_, has attracted considerable interest as a raw material for the production of biofuels and biochemicals via anaerobic fermentation processes such as syngas fermentation [1], microbial chain elongation [2], hydrogenotrophic methanation [3], and microbial assisted bioelectrochemical synthesis (BES) for conversion of CO_2_/CO [4]. Syngas can be generated via gasification of solid fuels such as coal and lignocellulosic biomass, which is an effective way of making use of the recalcitrant lignin present in lignocellulosic biomass [5,6]. In addition, CO is the major byproduct of incomplete combustion of carbonaceous materials such as coal, oil, and petroleum products, and it has been generated and discharged in significant quantities by related sectors such as steel industries [7]. Biological conversion of CO and/or CO_2_ and H_2_ into fuels and chemicals has many advantages over catalytic conversion (e.g., Fischer–Tropsch (FT) Synthesis), including higher product specificity, lower energy input, and increased resistance to poisoning by gas contaminants. The ability of certain types of microbes to utilize CO as their sole carbon and energy source or CO_2_ as their carbon source with their energy derived from CO or H_2_ following the Wood–Ljungdahl (WL) pathway has demonstrated tremendous potential for the production of different products such as acetate, ethanol, butyrate, and butanol, among others [8]. The WL pathway or acetyl-CoA biochemical pathway is the reductive synthesis of acetyl-CoA from CO_2_. The WL route is composed of two branches: methyl and carbonyl. The methyl (eastern) branch is involved in the conversion of CO_2_ into the methyl group of acetyl CoA. Whereas in the carbonyl (western) branch, CO_2_ is reduced to CO or CO is used directly from the medium to act as the carbonyl group for acetyl-CoA. However, during heterotrophic growth with sugars, the glycolysis pathway is linked to WL via pyruvate-acetyl-CoA reaction through the activity of enzyme ferredoxin oxidoreductase [9].

Due to the high miscibility of primary fermentation products such as short chain carboxylic acids (SCCAs) in their fermentation broth, energetic demands of the subsequent extraction and separation processes are very high. To circumvent this bottleneck, bioconversion of these SCCAs to less miscible organics such as medium chain carboxylic acids (MCCAs) through a process termed chain elongation via reverse β oxidation pathway is being identified as a potential alternative approach for recovery of resources from organic waste [10,11].

$$5C_{x}H_{2x-1}O_{2}^{-}+6C_{2}H_{6}O\to_{\;}5C_{x+2}H_{2x+3}O_{2}^{-}+C_{2}H_{3}O_{2}^{-}+4H_{2}O\;+H^{+}+2H_{2}$$

While ethanol and lactate are generally regarded as efficient electron donors for chain elongation, many other compounds such as methanol, sugars, CO, pyruvate, and hydrogen are also utilized [12]. At lower pH values, further reduction of MCCAs to corresponding alcohols can be accomplished by initiating solventogenesis [13]. MCCAs are primarily obtained from animal fats, plant oils or petroleum, and have wide industrial applications in manufactured products such as fragrances, pharmaceuticals, lubricants, etc. and as antimicrobial agents and food additives. MCCAs can be upgraded to jet biofuels [11].

Microbial fuel cells (MFCs) and microbial electrolysis cells (MECs) are bioelectrochemical systems (BESs) that have been widely researched for their ability to treat wastewater while simultaneously generating energy and hydrogen, respectively [14,15]. Other applications of bioelectrochemical technology include a combined approach for wastewater treatment, desalination of seawater/saltwater and energy production through microbial desalination cells [16], removal and recovery of nutrients [17] and metals [18]. In BESs, either anodic or cathodic or both redox reactions are catalyzed by microorganisms. In circumstances when the potential of the redox reaction catalyzed at the cathode is higher than that of the anodic, as in the case of oxidation of organics and presence of oxidant at the cathode, electricity is generated in MFC [19]. The anode-respiring bacteria (ARB) responsible for the reaction at the anode releases electrons, protons, and CO_2_. Whereas in MEC, under the absence of an oxidant at the cathode, current production is non-spontaneous, and an external power > 0.14 V is supplied in order to overcome the minimum potential of −0.4 V required for the generation of hydrogen upon reduction of protons at the cathode [20]. Nonetheless, the required voltage is substantially lower than that required for hydrogen generation through conventional water electrolysis (1.2–2.0 V) [21]. The requirement of a precious metal catalyst, such as platinum, on the cathode for hydrogen production and difficulty in hydrogen storage are considered disadvantages of this process. On the one hand, BES has been combined with dark fermentation/anaerobic digestion as a post-treatment process in order to increase the yield of a product from waste while also improving organic matter treatment [22]. On the other hand, BES-based technologies have remarkable potential for waste valorization, CO_2_ fixation, and the storage of renewable energy sources such as electricity and hydrogen in chemically stable forms as biofuels (methane, ethanol, butanol, etc.) and biochemicals (butyrate and caproate, etc.) The existence of microorganisms in BES that do not contribute to the metabolism of electroactive bacteria, on the other side, has a detrimental effect on the BES’s performance.

Electromethanogenesis is the process by which methanogens generate CH_4_ in MEC by converting CO_2_ either directly using electrons obtained from biocathode or indirectly with the in situ produced H_2_ from proton reduction, bioelectrochemically catalyzed by hydrogenase or electrochemically [23,24]. The quantity of methane generated through direct electron transfer is negligible, and the H_2_ generated in the latter process is used to reduce CO_2_ to methane via hydrogenotrophic methanogenesis under low cathodic potentials [24]. However, the electron transfer to microorganisms is also mediated via compounds such as acetate and formate produced by syntrophic microorganisms in methane-producing BES. In addition, direct interspecies electron transfer (DIET) has also been proposed for methane production in mixed culture [25]. Electroactive microorganisms present at the cathode either accelerate electron transfer to methanogens [26] or assist in the cathodic hydrogen evolution reaction [27].

$${\mathrm{CO}}_{2}+4H_{2}\to {\;\mathrm{CH}}_{4}+2H_{2}O\;\left(\mathrm{indirect}\right)$$

$$\;\;\;\;\;\;\;\;\;\;\;\;\;\;\;\;\;{\mathrm{CO}}_{2}+8H^{+}+8e^{-}\to {\;\mathrm{CH}}_{4}+2H_{2}O\;\left(\mathrm{direct}\right)$$

Methane can be generated electrochemically from CO_2_ at a theoretical voltage of −0.244 V (vs. SHE) at a pH of 7. However, due to the system’s various losses, a higher overpotential is required, which may be reduced by employing a microbial biocathode [24]. This one-step process offers many advantages over conventional multi-step anaerobic digestion, including higher CH_4_ yields and the possibility of effectively using anaerobic digestion waste such as CO_2_. Thus, integration of anaerobic digestion with BES technology presents a potential approach as a biogas upgrading technology for improving the methane content in biogas [28]. When feeding AD waste stream to the anode of a BES, the oxygen produced by water oxidation at the anode could aid in further hydrolysis of the residual organics, thus increasing overall methane productivity.

Through a process termed microbial electrosynthesis (MES), organics or CO_2_ can be transformed into multi-carbon compounds without undergoing methanogenesis in bioelectrochemical cells. Nevin and colleagues published the first data on CO_2_ reduction using this technique where the electrons transferred through graphite electrode to *Sporomusa ovate* producing acetate and 2-oxobutyrate [29]. The majority of published studies on CO_2_ reduction in MES have been limited to acetate as the main product, and improved production of chemicals such as ethanol, butanol, and butyrate have also been demonstrated as major products by manipulating pHs, poising the cathode with different potentials, and varying CO_2_ supply [30]. Use of photovoltaic or wind energy to generate electricity to power the MES for CO_2_ conversion to MCCAs and corresponding alcohols could eliminate the need for an external supply of electron donors such as H_2_ or CO in syngas fermentation and the need for external supply electron donors such as ethanol or lactate in the chain elongation process. This may pave the way for a more sustainable approach for carbon capture and utilization (CCU).

This review aims to provide the reader with a comparison and discussion of the different bioreactors utilized for biological and bioelectrochemical gas conversion. Parameters affecting the performance of these processes are included, such as biofilm development and gas–liquid mass transfer. Special attention is dedicated to providing the techno-economical assessment and life cycle analysis of gas fermentation. In addition, the extent to which these bioprocesses have been industrialized has been discussed.

## 2. Bioreactor Systems: Syngas Fermentation

**Figure 1:**
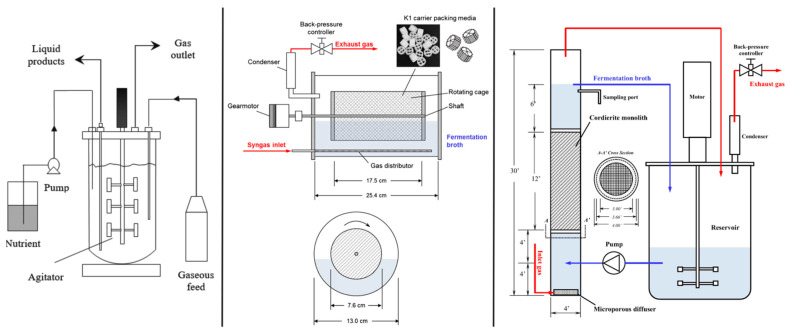
(**Left**) Stirred tank bioreactor (STB) reprinted from [39] (Copyright 2011), with permission from the Society of Chemical Industry and John Wiley and Sons Ltd. (**Middle**) Rotating packed bed biofilm reactor. Reprinted from [35] (Copyright 2017), with permission from Elsevier. (**Right**) Monolithic biofilm reactor. Reprinted from [38] (Copyright 2014), with permission from Elsevier.

Based on their growth mode, biological processes are categorized into two categories: suspended (planktonic) growth and attached (biofilm) growth. Microbes grow in bulk liquid medium in the suspended system, unattached to the packing materials. By far the majority of C1 gas fermentation investigations have been conducted in stirred tank reactors (STRs) (Figure 1), where gas–liquid mass transfer can be improved by increasing the impeller speed, which splits the gas stream into smaller bubbles with a larger interfacial area. However, for larger reactors, the requirement for a high power input to ensure a high gas–liquid mass transfer is uneconomical [31]. On the other hand, microspargers have mostly been employed in STR as a gas delivery device for the purpose of generating microbubbles.

Attached biofilm reactors bring a number of advantages over suspended biofilm reactors, including the ability to achieve a high biomass concentration, a compact footprint, a small reactor volume, and low energy requirements [32]. Through microbial immobilization, a high concentration of active biomass is obtained, which could improve the overall performance of the bioconversion system. However, higher density of cells also hinders the diffusion of gas into biofilm. Biofilm systems are further classified into fixed and moving carrier medium systems. In the first system, the carrier medium is stationary and the biofilm is attached to it; in the latter system, the biofilm-containing media is continuously moving inside the bioreactor through mechanical, hydraulic or pneumatic force. Biotrickling filter and biofilter are examples of a fixed carrier medium system. Rotating biological contractors (RBCs), moving bed biofilm reactors (MBBRs), and fluidized bed biofilm reactors (FBBR) are all examples of moving carrier medium systems [32,33]. Membrane fouling is a phenomenon that impairs the system’s performance. Membrane cleaning through gas sparging and scouring with granular activated carbon (GAC) are two methods being used to control fouling [34].

### 2.1. Rotating Packed Bed Biofilm Reactor

The carrier media are held inside an enclosed cage that is partially immersed in the liquid medium and partially exposed to the headspace gas. The cage rotates constantly, allowing the biofilm to grow on the carrier by alternately contacting gaseous and liquid phases. This results in the absorption of gaseous substrate from both headspace and liquid medium. When rotated and positioned with the headspace, a thin layer of liquid covers the biofilm, allowing efficient mass transfer from bulk gas to the cell surface by maintaining a high concentration of the gas-carried substrate. In this reactor system, the main rate-limiting step is diffusion across the gas–liquid interface [35]. Due to the cage spinning at a very low rate (3–60 rpm), energy consumption is significantly decreased, facilitating the scale-up process. However, maintaining an optimum rotation not only increases mass transfer but also helps prevent biofilm detachment. In a syngas fermentation study, a horizontal rotating packed bed reactor (Figure 1) demonstrated superior performance than a CSTR reactor, with a 3.3-fold increase in ethanol titer and productivity [35].

### 2.2. Monolithic Biofilm Reactor

The monolithic biofilm reactor (MnBR) may be regarded as an upgraded form of the bubble column reactor, as it contains a monolithic structure packed inside. This helps to prevent biomass washout at greater dilution rates because the whole design of the monolith is composed of a series of straight and parallel channels separated by thin and porous walls. The frictional forces of the flow of fluids can be reduced. Other promising features of monolith architecture include a large pore size and specific surface area, great mechanical strength, and ease of scaling up. They are typically made from cordierite (Mg_2_Al_4_Si_5_O_18_) or a silica-alumina compounds [36]. Under a particular flow regime, a thin layer of liquid slugs that forms between the gas bubbles and biofilm, flowing in a plug flow pattern inside the channels, along with internal recirculation of liquid slugs, allows achieving better mass transfer than a bubble column. However, there are different flow patterns observed in the monolith depending on the flow rate of fluids, channel geometry, and properties of fluids, etc. [37]. When the gas velocity is extremely low, a bubbly flow pattern is seen, resulting from the tiny bubbles that do not coalesce, making the monolith behave like a bubble column reactor. Shen et al. (2014) compared MnBR (Figure 1) with BCR for syngas fermentation and found that MnBR outperformed BCR by more than 50% in CO utilization efficiency (%), CO consumption rate (mmol/L/day), ethanol concentration (g/L), and ethanol productivity (g/L/day) [38].

### 2.3. Membrane Bioreactor (MBR)

**Figure 2:**
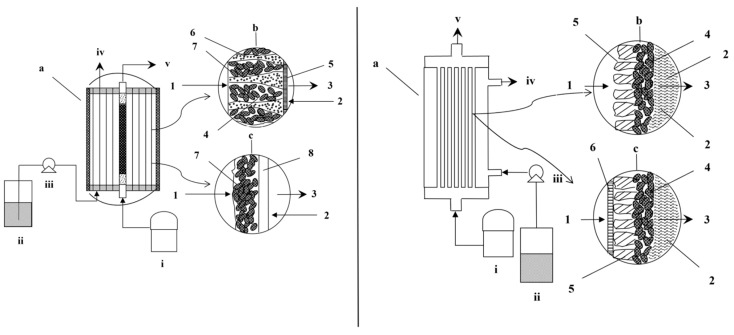
(**Left**) Membrane bioreactor (MBR) with gas fed through the outer surface of the membrane fibers and the liquid flowing through the hollow fiber lumens. (**Right**) Membrane bioreactor (MBR) with gas fed through the hollow fiber lumens while the liquid flows through the outer surface. The description of the figures can be found elsewhere [39]. Reprinted from [39] (Copyright 2011), with permission from the Society of Chemical Industry and John Wiley and Sons Ltd.

Membrane bioreactors are often used to treat the poorly water-soluble gases. Membranes may be made from microporous, dense, or composite materials with variable selectivity, mechanical strength, and permeability characteristics. In an MBR, waste gas is introduced through the membrane’s lumen and diffuses to the shell side, where microorganisms in the biofilm attached to the membrane surface degrade or convert these pollutants (Figure 2) [40]. Microporous hydrophobic membranes have high gas permeability while also acting as a barrier, preventing liquids from passing across the membrane. However, concerns over increased mass transfer resistance caused by membrane wetting and biofouling limit its widespread usage. The cost and durability of the membranes are other challenges of this technique. A composite membrane comprising a microporous hydrophobic membrane coated by a thin layer of dense material not only provides a better interface but also reduces biofouling [40,41].

A hollow fiber membrane biofilm reactor made of microporous polypropylene (PP) was used for syngas fermentation utilizing *Clostridium carboxidivorans* P7 attached to the membrane and demonstrated a maximum ethanol production of 23.93 g/L in continuous mode operation [42]. With the reactor configuration, a high dilution rate of 0.96 day^−1^ was achieved without biomass being washed away, which is double the rate achievable with a suspended culture system employing the same strain [42]. The ability to operate at a higher dilution rate supplies microorganisms with a nutrient-rich environment that promotes growth while also facilitating acidogenesis. At a lower dilution rate, however, the microbial metabolism may be switched to solventogenesis. Thus, the overall syngas fermentation performance by this bioreactor configuration has the potential to outperform suspend-growth bioreactors.

### 2.4. Moving Bed Biofilm Reactor (MBBR)

**Figure 3:**
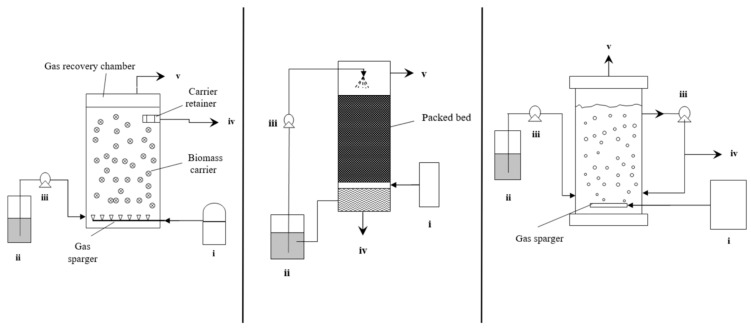
(**Left**) Moving bed biofilm reactor (MBBR). (**Middle**) Trickling bed reactor (TBR). (**Right**) Bubble column reactor (BCR). i—Gaseous feed; ii—Nutrient feed; iii—Pump; iv—Liquid products and v—Gas outlet. Reprinted from [39] (Copyright 2011), with permission from the Society of Chemical Industry and John Wiley and Sons Ltd.

By using free-floating carriers with attached microorganisms, the benefits of both activated sludge and biofilm reactors are combined in a moving bed biofilm reactor (MBBR) (Figure 3). The problems associated with trickle bed or membrane reactors such as clogging and channeling could be potentially alleviated by this technology [33]. Additionally, this configuration has benefits during syngas fermentation, such as the possibility to enhance the concentration of slowly growing anaerobic bacteria by delivering more carriers, and the ability to facilitate fluidization of the carrier when feeding the syngas into the system. Numerous factors, including the biofilm, the carrier medium and its characteristics, and liquid and gas flow rates, all contribute significantly to the performance of MBBR. MBBR technology has been primarily utilized in the treatment of wastewater from various industrial sources for the purpose of nutrient removal and resource recovery [43]. However, its application in syngas fermentation is not well documented. A recent study conducted in a high pressure MBBR outperformed the high-pressure suspended culture reactor by 33% in terms of H_2_ uptake and 48% in terms of acetic acid production rate [44]. However, in that study, a mechanical stirrer was employed to fluidize the carriers.

### 2.5. Trickle Bed Reactor (TBR)

Trickle bed reactors have packing materials providing a large volumetric surface area for microorganisms to attach and develop biofilms for the biochemical reactions (Figure 3). Within the reactor, the gas and liquid phases are supplied either co-currently or in countercurrent directions. By employing a trickle feeding approach, necessary nutrients can be supplied to the microorganisms while simultaneously reducing resistance to gas–liquid mass transfer by forming a thin layer of liquid film [45]. In contrast to CSTR, which is heavily reliant on mixing for gas–liquid mass transfer, TBR does not require mechanical agitation. Additionally, unlike CSTR, TBR allows for independent control of the superficial gas velocity. However, inconsistent irrigation of packing materials results in the formation of stagnant zones devoid of microbial activity, resulting in a reduction in overall performance. According to research conducted on TBR by Devarapalli et al. (2017), switching from countercurrent to co-current mode led to a maximum ethanol production and productivity of 13.2 g/L and 158 mg/L·h by *Clostridium ragsdalei* using 6 mm soda lime glass beads (void fraction of 0.38) as packing material, while also alleviating flooding issues [46]. This ethanol productivity rate is four times higher than that of a semi-continuous TBR operation studied by the same research group [47].

### 2.6. Bubble Column Reactor (BCR)

A bubble column reactor (two-phase or slurry) is a vertical cylindrical vessel that holds the liquid phase and is supplied with gas from the bottom. It has several operational advantages over other bioreactor designs, including low maintenance and operating costs due to the absence of mechanical mixing components, as well as superior heat and mass transfer performance (Figure 3) [48,49]. The reactor may operate in semi-continuous mode, with the liquid phase introduced in batches, or in continuous mode, with the liquid phase introduced co-currently or counter-currently with the upward flow of gas. The length-to-diameter ratio of the column, or alternatively known as the aspect ratio, is typically between two and five for biochemical applications [48]. Additionally, many hydrodynamic parameters such as gas holdup, axial dispersion coefficient, liquid phase properties and others should be considered while designing a BCR. The gas–liquid flow regimes changed from homogeneous (bubbly flow) to heterogeneous (churn turbulent flow, slug flow, and annular flow) regimes as the gas velocity increased [50]. On the other hand, an airlift reactor is a kind of bubble column reactor that was designed to enhance flow circulation with a defined liquid flow pattern. It has two interconnected compartments: the riser receiving the gas stream, and the downcomer containing only a small amount of the gas phase. Due to the density differential between the riser and downcomer, liquid circulation occurred. It is available in two types: internal loop and exterior loop. The reactor zone is separated in an internal loop airlift reactor by a draft tube or split cylinder. However, in an external loop airlift reactor, horizontal segments link the vertical tubes at the top and bottom [51]. As the pressure decreases with height, the bubble size gradually increases with the length of the column, affecting the mass transfer of the gaseous substrate. Richter et al. (2013) performed syngas fermentation by *Clostridium ljungdahlii* in a two-stage continuous system comprised of a 1-L CSTR as the growth reactor and a 4-L bubble column connected with a membrane module as the ethanol production reactor, achieving an ethanol productivity of 0.374 g/L.h in the ethanol production reactor. A microbubble sparger with pore size of 0.5 μm was used to diffuse the syngas into the column [52]. Rajagopalan et al. (2002) used a fritted glass disc of pore size between 4 and 6 μm at the base of the 6.2-L bubble column for feeding syngas for the fermentation by *C. carboxidivorans* (strain P7) [53]. Shen et al. (2014) reported an ethanol productivity of 1.54 ± 0.30 g/L/day in a BCR fed by syngas through two wooden 50-µm microporous diffusers at the base of the reactor [38].

## 3. Bioreactor Systems: Microbial Chain Elongation

Careful selection of waste management systems, unit activities, and raw materials will facilitate the transition from fossil to bio-based economies. Selecting any process’s end product as a starting material for subsequent bioprocessing would help to advance the biorefinery approach. The WL pathway has the potential to result in the synthesis of ethanol and acetic acid from syngas fermentation [54]. Since the concentration of ethanol produced from syngas fermentation is lower than that obtained from existing ethanol production methods, the cost of product separation is substantially greater than that of conventional ethanol production. As a result, new platform chemicals such as caproate, and caprylate, which are less soluble in water and have a high market value (<2 €/kg), are preferred. These MCCAs have a wide range of applications such as antimicrobial, anticorrosion agent, plasticizers, etc. [12]. The significance of microbial chain elongation is growing as strains such as *Clostridium kluyveri* have shown their ability to convert acetate and ethanol to short or medium chain fatty acids [55,56]. Temperature/pressure, product selectivity, and energy expenditure are critical factors for chain elongation [57]. These parameters can be optimized by using different types of bioreactors.

For odd-chain elongation, such as even-chain, the chain elongating microorganisms use a reverse β oxidation metabolic pathway. During odd chain elongation, ethanol is first converted to acetyl-CoA, which later combines with propionyl-CoA to yield valerate. Likewise, valerate is converted to heptanoate. However, in every five chain elongation reaction steps, one molecule of acetyl-CoA is used for the ATP generation using substrate level phosphorylation by converting it to acetate [58,59]. Therefore, part of the ethanol is anaerobically oxidized to acetate, giving the chain elongating microorganisms the possibility to produce even chain fatty acids such as butyrate and caproate. Subsequently, in most of the odd chain elongation bioprocessing, product distribution extended towards the generation of even chain carboxylates. One of the process parameters that controls the final end product concentration in odd chain elongation is the ratio of ethanol:propionic acid. It was observed that keeping this ratio low will help to improve the ethanol efficiency for reverse β oxidation from oxidation to acetate, resulting in diverting the product spectrum more towards odd-chain fatty acids [58].

During heptanoate production from propionate elongation, the presence of acetate diverts the elongation towards caproate production rather than towards heptanoate. Furthermore, the authors observed less effect on ethanol load enhancement on heptanoate selectivity. It is speculated that chain elongation of propionate using proponal as an electron donor resulted in the production of more caproate. However, a maximum heptanoate production of 3.2 g/L with a selectivity of 23% was achieved in an up-flow anaerobic filter by using a mixed culture [60].

Utilization of substrates such as OFMSW for MCFA production proceeds through hydrolysis and acidification before chain elongation steps. High amounts of MCFA in their undissociated form inhibit the whole process. One way to overcome this inhibitory effect on microorganisms is by keeping the pH higher during acidification. This will allow the MCFA to be present in their dissociated form, which is less toxic. On the other hand, integrating an in-line extraction system is a plausible solution to enhance the final MCFA titer. However, a two reactor system that separates the hydrolysis/acidification from chain elongation steps will eliminate the inhibitory effect of MCFA and ethanol on hydrolysis. A downside of this approach is that increasing the pH to reduce the toxicity of produced MCFA could result in accelerating the activity of methanogens [61].

Grootscholten et al. (2013a,b) observed an increase in medium chain fatty acid productivity from acetate and ethanol in an up-flow anaerobic filter by reducing the hydraulic retention time. This is important while using mixed culture as low HRT will help to washout suspended acetotrophic methanogens, which compete with chain elongating microbes for acetate resulting in reduced MCFA production. In their studies, an MCFA selectivity of more than 80% (mol e eq/mol e eq × 100%) throughout their experimental run was achieved, although they take into account the electrons from yeast extract [62], [63]. However, with the lowest HRT of 4 h, the obtained concentrations of caproic acid (9.3 g/L) and caprylic acid (0.3 g/L) were lower than the solubility of their carboxylic acid forms in water, making their separation harder [62]. Some of the ethanol is oxidized, producing acetate and hydrogen. However, increased hydrogen may limit the growth of chain elongators such as *Clostridium kluyveri* [64].

A high specific exchange surface area enables a high volumetric gas transfer rate in hollow fiber membrane bioreactors (HFMBR), which improves production rates and lowers investment costs. The primary benefit of this kind of reactor is its resistance to microorganism washout. Although the amount of research on fatty acid synthesis utilizing these systems is limited, it is regarded as a critical technology for MCCAs production. Chain elongation was used to produce MCFA in situ from H_2_ and CO_2_. The concentrations of acetate, butyrate, caproate, and caprylate were 7.4, 1.8, 0.98, and 0.42 g/L, respectively. A mixed culture microbial community study revealed a predominance of *C. ljungdahlii* and *C. kluyveri* [65].

San Valero et al. (2020) investigated five distinct parameters for increasing hexanoic acid production in a CSTR. These parameters are critical factors for the effective operation of bioreactors for the production of MCCAs, including the use of inorganic carbon sources such as biocarbonate, the presence or absence of yeast extract, the ethanol content, and the pH. The microbial chain elongation of acetic acid, butyric acid, and ethanol to hexanoic acid utilizing *C. kluyveri* was carried out. The authors investigated several pH levels (7.5, 6.8, and 6.4) and observed up to 19.4 g/L hexanoic acid synthesis. The beneficial impact of adding an inorganic C source raised the concentration of hexanoic acid to 21.4 g/L [66].

The most often utilized method for MCCAs synthesis is the combination of two reactor systems, with one producing acetate and ethanol and feeding those products to another reactor for the chain elongation process. Nonetheless, using the same reactor and operating directly on gaseous substrates is much more preferable. Co-culturing of acetogenic *Clostridium* spp. with *C. kluyveri* is one method for MCCAs synthesis in batch reactors [67,68]. Co-culturing using syngas (60% CO, 35% H_2_, and 5% CO_2_) as the gaseous substrate and CO + H_2_ as the electron donor was investigated utilizing a 2 L Chemostat (CSTR) with a working volume of 1 L to generate caproate. *C. ljungdahlii* was employed to generate acetate and ethanol, whereas *C. kluyveri* was utilized for chain elongation in the same reactor with co-culturing, yielding 70 mmol C/L/day of caproate by using in-line product extraction. Longer chain alcohols such as *n*-hexanol, and *n*-octanol were produced at a rate of 31.7, and 0.045 mmol C·L^−1^·d^−1^, respectively, by the action of *C. ljungdahlii* [69].

**Figure 4:**
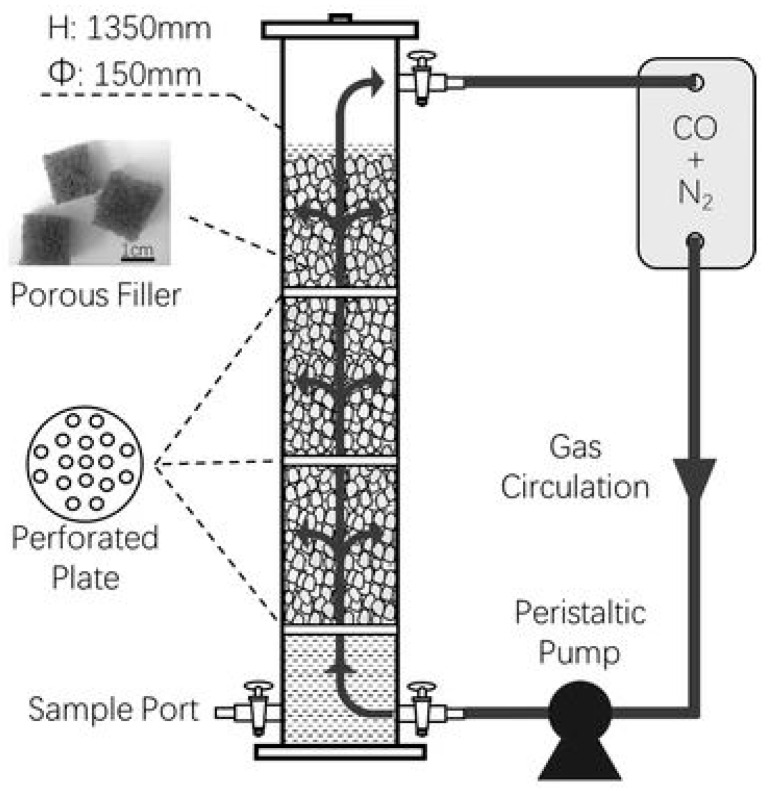
Schematic of the reactor used for carboxylates production from CO using open culture by He et al. (2018). Reprinted from [70].

While the increased MCFA yields and decreased energy requirements are advantages of combining two cultures, the sterilizing requirements and stability challenges associated with working with pure cultures are the primary drawbacks. To overcome these challenges, the feasibility of utilizing mixed cultures for MCFA production was also investigated. He et al. (2018) investigated using a 21-L polymethyl methacrylate reactor with an 18-L working volume running in semi-continuous mode. The reactor was filled with small cubic polyester fibers with a high surface area to support anaerobic mixed culture immobilization (Figure 4). The reactor was supplied with CO, which also acted as a methanogen inhibitor, and concentrations of n-caproate, n-heptylate, and n-caprylate 1.892, 1.635, and 1.033 mmol/L, respectively, were produced [70].

## 4. Bioreactor Systems: Hydrogenotrophic Methanation

**Figure 5:**
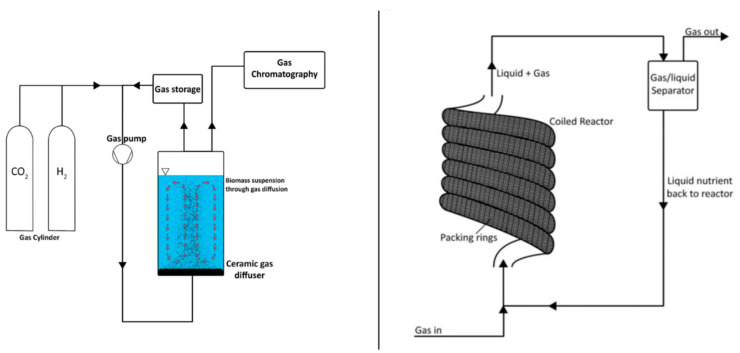
(**Left**) Diffusion based reactor. (**Right**) Tubular loop reactor made of a. narrow diameter pipe and packed, fixed biofilm. Reprinted from [71] (Copyright 2019), with permission from Taylor and Francis Group.

Methane production from CO_2_ has been shown to be a feasible process using H_2_ as an electron donor. CSTRs, diffusion-based reactor systems, fixed bed reactors, minimum liquid bioreactors, fixed film reactors based on soil, and hollow fiber reactors were all operated for hydrogenotrophic methanation (Figure 5) [71]. CSTRs are the most widely utilized reactors in which the gas substrate is exposed to agitation. The critical factor for the processes is the size of bubbles used to transport gas into the bacterium. Rusmanis et al. (2019) stated in a comprehensive review of hydrogenotrophic methanation that the methane evaluation rate varies between 0.86 L CH_4_/L/d and 800 L CH_4_/L/d on an experimental to industrial scale [71].

Biofilm forming reactors have been developed and are extensively utilized to circumvent the mass transfer constraints of gaseous substrates. Immobilization of anaerobic microbes on a support material or the use of biofilm systems, for example, has been shown to increase production efficiency. Trickle bed reactors have been utilized in this regard for biologically catalyzed methanation. Burkhardt and Busch (2013) obtained a production rate of 1.17 Nm^3^CH_4_/m^3^·d using a patented trickling bed reactor with a fixed bed capacity of 26.8 L and a process water volume of 5 L, loaded with Bioflow 40 immobilization material [72]. Similarly, Strübick et al. (2017) utilized a 58.1 L trickling bed reactor operating at a thermophilic temperature of 55 °C. With 98% methane concentrations, a 15.4 m^3^CH_4_/m^3^·d methane production rate was recorded [73]. Dupnock and Deshusses (2017) developed a bench-scale PVC tubular biotrickling filter. The reactor was packed with polyurethane foam and inoculated with hydrogenotrophic methanogens. From CO_2_ and H_2_ (20:80% vol.) feeding, a maximum methane output of 38 m^3^CH_4_/m^3^·d was recorded. DNA sequencing revealed that *Euryarcaeota* accounted for 27% of the biomass. It was demonstrated that optimizing biomass density and activity may result in higher biogas upgrading rates [74].

Using biochar as a biocarrier is one of the innovative approaches for increasing methane production rates. The black ceramsite and biochar made from corn straw and digestate were utilized to assess the bioconversion of CO_2_ to methane. The addition of carrier materials increased the methane production rate by 20%, while the addition of corn straw biochar and digestate biochar could even increase the rate up to 70% compared to suspension culture [75]. Daglioglu et al. (2021) have also shown the beneficial impact of immobilization in a comparative study utilizing glass pipe and ceramic ball. The use of glass pipe and ceramic balls as immobilization medium resulted in methane production rates of 4.8 and 3.9 m^3^CH_4_/m^3^·d, respectively [76]. Increased methane production rates may be possible because of the longer retention periods for methanogenic biomass.

Another option for hydrogenotrophic methanation is by using membrane biofilm reactors. A membrane biofilm reactor with a pseudo-dead-end for ex-situ biogas upgrading utilizing biogas as the only carbon source was investigated by Miehle et al. (2021) [77]. The reactor comprised a bundle of 19 submerged, dead-end and hydrophobic polypropylene tubular membranes (surface area 0.198 m^2^) placed within a stainless steel module. By feeding a H_2_: CO_2_ of 4:1, 2.21 v v^−1^ d^−1^ space-time yield, which is methane volume produced per reactor volume and day, was able to be achieved. Protofiorito and coworkers [78] produced a maximum methane production per reactor volume of 1.17 Nm^3^/m^3^ with a 97 percent methane content utilizing a custom-made membrane biofilm reactor with a membrane surface to reactor volume ratio of 57.9 m^2^/m^3^. The authors predicted that productivity might be increased to 12 Nm^3^ m^−3^ d^−1^ by employing a system with a 600 m^2^/m^3^ specific membrane surface by using capillary or hollow fiber membranes with a considerably smaller diameter.

Another successful strategy for increasing methane production rates is by addition of nanoparticles. Fe nanoparticles were utilized to increase the abundance of *Methanothermobacter* in methanogens, thus increasing the efficiency of CO_2_ and H_2_ conversion to CH_4_. The 16sRNA gene sequencing study revealed an increase in *Methanothermobacter* abundance from 7% to 16%. The methane production yield increased significantly from the 0.105 to 0.186 L/L reactor [79].

Concerning all kinds of hydrogenotrophic methanation systems, a set of parameters and system boundary definitions should be defined in order to unify and standardize the data collected for evaluating the efficiency of various systems. Thus, a methanation system was proposed in order to determine its efficacy in terms of performance, biology, and cost by Thema et al. (2019). Additionally, a standardization for various data display units was presented as well [80].

Table 1 provides the merits and drawbacks of different bioreactor configurations for C1 gas fermentation. The major obstacle to C1 gas fermentation is the gas mass transfer inside the bioreactor system and one way to address this is by elevating system pressure [81]. However, working at elevated pressure was found to have a significant effect on changing the product spectrum [82].

## 5. Bioelectrochemical C1 Gas Conversion

Bioelectrochemical systems (BES) are often utilized in renewable energy production [83], wastewater treatment [84,85], nutrient recovery [17], biosensing [86], and bioremediation applications [87]. Electroactive bacteria in BES are capable of transporting electrons from or to their cells to the extracellular environment and forming biofilms on electrode surfaces [86,87,88]. A recent and very promising use of BES is the electrical stimulation of cellular metabolism, which directs electron flow to the desired products through a process termed electro-fermentation [89]. Electro-fermentation has the potential to significantly improve the efficiency of microbial catalytic activity by electrochemically controlling the microbial fermentative processes with electrodes and providing additional electron donors or acceptors to the cells in order to balance the fermentation [89,90]. *Clostridium pasteurianum*, for example, generates butanol and 1.3-propanediol through electro-fermentation utilizing glycerol as a carbon source [91]. Among all anodic and cathodic routes, the conversion of CO_2_ to value-added products intrigued BES researchers owing to its novelty, environmental significance, and industrial potential [92]. Microbial electrosynthesis, or bioelectrochemical synthesis, is a process in which CO_2_ is reduced by electroactive biofilms using electrons derived from BES cathodes [93]. In bioelectrochemical synthesis, valuable chemicals such as methane [24], acetate [29], butyrate [30], and ethanol [94], among others, are synthesized using electricity.

**Figure 6:**
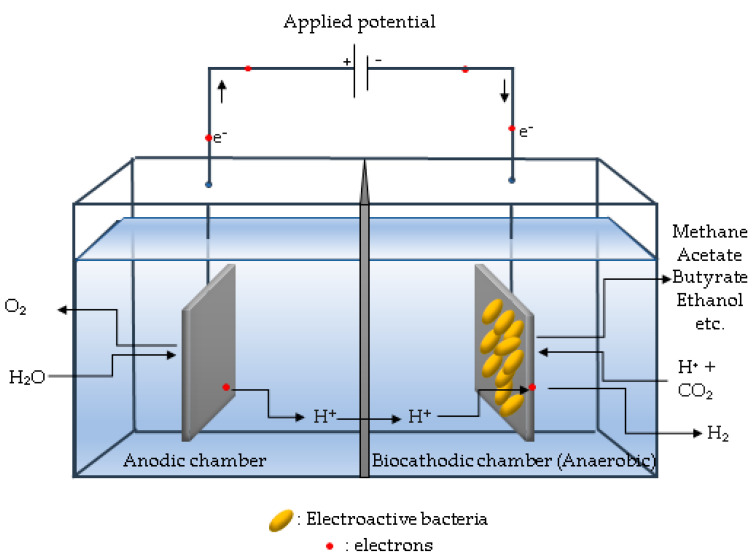
Bioelectrochemical system for CO_2_ reduction to chemicals and fuels.

The configuration and design of MES, such as BES, in general requires knowledge of a wide range of scientific fields, including microbiology, electrochemistry, materials science, environmental engineering, and biological engineering. A conventional BES consists of an electrochemical reactor with a membrane between the anodic and cathodic compartments (Figure 6). An external power source is used to link the anode and cathode. Biofilms colonize either on the anode or on the cathode, which are referred to as bioanode or biocathode, respectively.

Electrodes can be constructed from a wide range of materials. Carbon is the most versatile material, which is available in compact form as graphite in plate, tube, or granule form, in fibrous form as filters, cloth, paper, fibers, and foams, and as brushes and glassy carbon [95,96,97,98]. Increased surface areas are accomplished by using compact materials such as reticulated vitreous carbon, which comes in many different pore sizes and can be utilized in layers [99]. The high porosity of materials is critical in preventing fouling [100]. The electrode materials must be electrically conductive, preferably highly conductive, biocompatible, chemically stable in the reactor solution, non-fouling, and non-corrosive. They have a high specific surface area (area per volume) adapted for the growth of the biofilm, which will be responsible for the majority of the electron transfer, and have the capacity to promote sufficient turbulence for proper proton diffusion between the membrane and the opposite electrode; in addition, they are inexpensive and simple to make and scale to larger sizes [99,100]. Different strategies can be used to improve the performance of electrodes, including the incorporation of chemicals such as Mn (IV) and Fe (III) into the electrode structure [101], the deposition of carbon nanotubes [102], and the use of exogenous artificial mediators [100].

In MES, the cathode stands out for being the support and electron donor. Thus, the cathode needs to be biocompatible, highly conductive, and exhibit long-term stability [92]. The ambition of industrial application requires that it should be inexpensive (devoid of precious metal catalysts), yet with adequate performance for H_2_ evolution in non-ideal electrolytes [102]. The cathode’s porosity must be balanced between higher projected current density and microbe immobilization (thicker electrodes) and a more efficient mass transfer and lower ohmic drop (thinner electrode) [92]. Gas diffusion cathodes could be chosen to decrease the CO_2_ mass transfer limitation [92].

The majority of BES designs need a physical separation of the anodic and cathodic compartments. Membranes are mainly employed in two BES chambers to maintain the separation of the anolyte and catholyte. These membranes must allow protons generated at the anode to pass through. Additionally, membranes act as a barrier to undesirable substrate flow from the anode to the cathode (fuel cross over). The most often used cation exchange membrane (CEM) is Nafion (Dupont Co., USA), which is available in a variety of thicknesses, with 117 being the most popular [100]. Ultrex CMI-7000 (Membranes International Inc., NJ, USA) is a substitute for CEMs [103], with a more favorable relative cost/effectiveness than Nafion [100]. Kim et al. (2007) demonstrated increased efficiency using the anion exchange membrane (AEM) AMI-7001 (Membranes International Inc.) [104]. Furthermore, bipolar, anion, and cation membranes may be utilized [105]. The drawbacks of membranes in BES include their high cost (Nafion 117 may cost up to EUR 2800 per m^2^) and the system’s performance decreases as internal resistance increases. In order to avoid biomass clogging while also allowing relevant mass transfer (for H_2_, CO_2_, organic products, and alkalinity) at high current densities, the distance between the cathode and the membrane should be kept to a minimum [92,106].

## 6. Reactor Systems: Bio-Electrochemical Synthesis

Numerous parameters, including the substrate type (gaseous such as CO_2_, CO, or dissolved compounds), the targeted products and their recovery (gaseous or dissolved compounds), and the separator requirements (membrane or membraneless), must all be considered when designing a bioelectrochemical reactor (BER), as these factors will have a significant impact on the process’s performance and economics. The most significant limiting variables influencing BER performance are ohmic loss, concentration polarization, and electrode overpotential. All of them must be minimized by the reactor design [107]. The Ohmic loss can be minimized by shortening the distance between the anode and cathode, employing a high conductivity electrolyte, and utilizing a low resistivity separator [108]. The concentration polarization is caused by the difference in the rate of reactions at the electrodes during short supply of substrate and the rate of migration of ions between the electrode surface and the electrolytes. This mass transport process is affected and controlled by diffusion, migration (electric field) and convection velocity [109]. Although electrode overpotential is primarily determined by the catalytic property of electrode, an increased electrode surface-to-volume ratio, which must be considered during reactor design, may help decrease the loss [108].

### 6.1. Single-Chamber Bioelectrochemical Reactor

In this bioreactor configuration, both anode and cathode electrodes are located in a single compartment without a membrane and using a single electrolyte. Thus, a membrane-free reactor may potentially decrease mass transfer resistance, resulting in higher current densities and cost savings owing to the absence of a membrane. However, since the system is entirely anaerobic, methanogens will consume the hydrogen generated at the cathode, forcing the electroactive bacteria to compete with methanogens for substrates such as acetate and product hydrogen. By covering the cathode side with a plate to restrict oxygen entry, a single chamber air cathode used in MFC mode and used to enrich electroactive bacteria can be changed to function as MEC. Katuri et al. [34] developed a tubular anaerobic electrochemical membrane bioreactor (AnEMBR) by combining MEC and nickel-based hollow-fiber membranes for energy recovery in the form of biogas from a low-strength solution (300 mg/L COD). The nickel-based hollow-fiber membranes acted as a cathode for hydrogen evolution while also filtering the effluent. According to reports, this nickel-based hollow fiber membrane was 70 times less expensive than a platinum-catalyzed electrode. The estimated net energy requirement is 0.27 kWh/m^3^ for a reactor operating at 0.7 V. Cheng et al. [110] developed a novel membraneless rotatable bioelectrochemical contactor (RBEC) to address pH shift and the occurrence of high cathodic overpotential in systems that use selective ionic membrane separators. Multiple spinning discs are joined on a shaft, rotate intermittently in this design, with the top and bottom discs physically and electrically isolated. The evenly developed biofilm on the disc’s surface catalyzes the anodic reaction when the half-disc is exposed to submerged liquid, while the other upper half-disc exposed to the gas phase catalyzes the cathodic reaction, generating methane and hydrogen. The pH split phenomena by creating acidification and alkalization in distinct compartments of BES has been exploited for product extractions, concentrations, and nutrient recovery [111]. Guo et al. (2010) demonstrated a high hydrogen recovery rate using a cathode-on-top single chamber configuration (Figure 6) [112]. However, to reach such a rate, an applied voltage greater than 0.5 V (up to 1 V was tested) was required. With a smaller cathodic chamber on top of the anodic chamber, the authors were able to easily limit and collect hydrogen without going through the anodic side, which contains microorganisms. The anode electrode was graphite granules, and the cathode was a mipor titanium tube coated with platinum, both of which were put within a glass reactor with a 30 mm distance between the electrodes. Internal resistance caused by the distance between the anode and cathode electrodes was found to cause a low hydrogen production rate. Furthermore, the cathode-on-top arrangement was tried using a fluidized bed of granulated activated carbon (GAC) as the anode. The cathode used was a cylindrical stainless steel mesh. By fluidizing the material, it is possible to overcome constraints such as insufficient electrical contact between the GAC and the current collector, as well as issues associated with biomass clogging. This column type reactor with a single chamber (empty bed volume of 40 mL) is called the “microbial fluidized electrode electrolysis cell (MFEEC)” and has been tested for hydrogen generation by Liu et al. [113]. At a liquid circulation rate of 17 mL/min, the hydrogen yield from acetate was increased by 116% when compared to the control without the GAC addition. By optimizing the anode configuration, which included two anodes (graphite felts) on each side and a cathode (carbon cloth) in the center of the single chamber cube type MEC, a hydrogen production rate of 10.88 m^3^/m^3^d was achieved at a 1.5 V applied voltage. In comparison to the conventional electrode arrangement, which places the anode on one side, this stacking anode arrangement boosted the hydrogen production rate by 118% at 0.8 V applied voltage [114].

### 6.2. Tubular Bioelectrochemical Reactor

**Figure 7:**
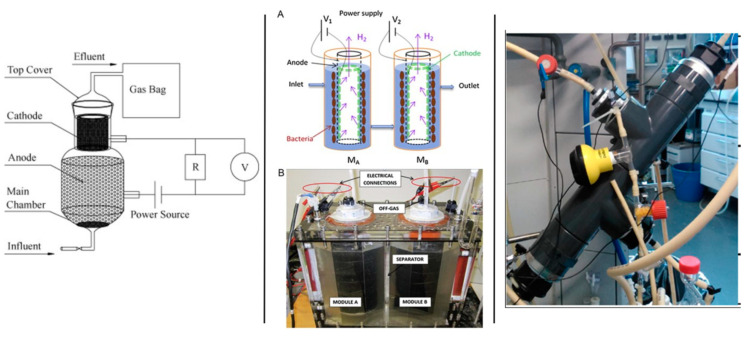
(**Left**) Schematic of the cathode-on-top single-chamber of the bioelectrochemical system [112]. (**Middle**) Pictorial view of semi-pilot tubular [A: Schematic view; B: Photographic view] [117]. (**Right**) Small scale tubular reactor for microbial electrosynthesis [30]. Reprinted with permission from Elsevier.

The associated scaling-up issue, such as optimizing the electrode and membrane surface area in proportion to the reactor volume, could be addressed easily by employing a tubular-type reactor [115]. Batlle-Vilanova et al. (2017) [30] constructed a tubular system with concentric inner and outer compartments, the former serving as the cathode (carbon cloth as electrode) and the latter as the anode (Ti-mixed metal oxide), separated by a tubular cation exchange membrane, for butyrate production from CO_2_ using the MES process at a cathode potential of −0.8 V (vs. SHE). The anode and cathode compartments had a net liquid volume of 1.49 and 1.30 L, respectively. To increase the synthesis of more reduced compounds such as ethanol and butyrate, a strategy of increasing hydrogen partial pressure by restricting CO_2_ input was used (Figure 7) [30]. Blasco-Gómez et al. (2019) utilized a reactor with a similar design for the production of acetate and ethanol, with a carbon cloth serving as the anode and a cathode chamber filled with granular graphite serving as the cathode [116]. Two single-chamber tubular MECs hydraulically linked in series for hydrogen generation have been tested using low strength household wastewater by Gil-Carrera et al., 2013 (Figure 6) [117]. Each MEC tubular module comprised an anode compartment that can accommodate up to 2 L of liquid and 0.2 milliliters of headspace. A vertical polypropylene tube with perforations was maintained in the center of the MEC to collect the generated gas. A nickel-coated gas diffusion electrode functioned as the cathode and was in contact with the outer surface of the polypropylene tube. To avoid a short circuit between the anode and cathode, an electric insulator (porous cellulosic nonwoven fabric) was placed over the cathode. On the other side of the insulator, an anode in two layers of carbon felt was maintained. As a current collector, a thick titanium wire was wrapped around the outside edge of the tubular anode. According to the findings of the research, when organic loading is less than 0.67 g-COD L/d, a single module MEC may compete with aerobic wastewater treatment in terms of energy consumption and treatment efficiency.

### 6.3. Dual-Chamber Bioelectrochemical Reactor

The H-type cells are the two most often utilized chamber cells. While this configuration is excellent for preliminary studies, it often exhibits a higher internal resistance owing to the greater distance between the anode and cathode. Vassilev et al., 2018 [118] examined the long-term operation of MES generating 4.9 g/L acetic acid, 3.1 g/L butyric acid, 1.6 g/L isobutyric acid, 1.2 g/L caproic acids, and corresponding alcohols 1.3 g/L ethanol, 0.8 g/L butanol, 0.2 g/L isobutanol and 0.2 g/L hexanol from CO_2_ in a modified glass vessel under semi batch mode at mild acidic pH conditions. In this design, the anode chamber is constructed by inserting a polyethylene tube into the graphite granule-filled glass vessel (cathode chamber). A tubular cation exchange membrane at the bottom of the tubes seals and physically separates the two chambers. In this MES, the working, reference and counter electrodes used are graphite granules, Ag/AgCl and platinum wire, respectively, poising the cathode potential at −0.8 V vs. SHE. The anode chamber contains the phosphate buffer solution and cathode chamber with the medium.

## 7. Biosensors in Bioelectrochemical Synthesis

Bioelectrochemical synthesis is a highly promising application of microbial electrochemical technologies for sustainable production of organic compounds. When waste treatment and valorization are included, it becomes even more significant, contributing to circularity. Monitoring the bioelectrochemical synthesis will allow to control and achieve greater efficiency from the process. Controlling these processes is often accomplished by regulating the potential of the reactions, but when microbes are involved, this may not be sufficient, resulting in poor reproducibility of the findings. Additional control is needed for industrial applications. This highlights the critical role of biosensors in controlling bioelectrochemical production, while also emphasizing the development of materials that are more sensitive and their miniaturization.

Biosensors can be used to precisely control the electro-fermentation either indirectly measuring the process conditions or measuring the presence of products, byproducts, biomass and or mediators [119]. The most common biosensors used industrially to control biotechnological processes are the commercial glucose biosensor or glutamate biosensor [120]. Another possibility is to use bioelectrochemical biosensors. Bioelectrochemical biosensors are straightforward to use, selective, affordable, and automatable, and they provide reproducible results [119].

In 2004, Heiskanen et al. developed a bioelectrochemical biosensor for real-time monitoring of living cells intracellular redox enzyme activity with a double mediator system. 2-Methyl-1,4-naphthoquinone (menadione, vitamin K3) and water soluble 2-methyl-1,4-naphthoquinone sodium bisulfite (menadione sodium bisulfite) were immobilized on platinum microband electrodes [121]. This method can be applied for assessing the redox state of NAD(P)H:quinone oxidoreductase, NAD(P)H oxidoreductase, NADH dehydrogenase in living cells. Sun et al. (2019) developed a three-chamber microbial electrochemical system as a biosensor for monitoring the acetate evolution during anaerobic digestion, which can also be used for acetate monitoring in BES [122]. An up-flow air–cathode chamber microbial fuel cell biosensor was also tested for in situ monitoring of biohydrogen and biomethane generation in bioreactors [123].

Additionally, bioelectrochemical biosensors can also be used for water toxicity assessment, namely the presence of 3,5-dichlorophenol (DCP) in water, using the electrochemical activity of *Shewanella oneidensis* MR-1 cells in a three-electrode system (platinum wire as the counter electrode, a saturated calomel electrode (SCE, +0.243 V vs. SHE) as reference electrode, and a carbon cloth (1 × 2 cm) as the working electrode) [124]. The presence of DCP in the water retarded the current evolution in BES, thus influencing the electro-fermentation processes.

## 8. Gas–Liquid Mass Transfer

Fermentation systems, including microbes and substrates in the gaseous state, face difficulties in terms of mass transfer from the substrate trapped in gas bubbles to liquid media or directly to the microbes, resulting in low cell density. The rate of mass transfer of these gaseous substrates is dependent on many factors, including the pressure exerted on the gas bubbles, the surface-to-volume ratio of the gas bubbles, and the gas bubble retention time [125]. C1 gas fermentation bioreactors operate in one of two regimes: mass transfer- or kinetic-limited. The former state occurs in those bioreactors where the mass transfer rate of these sparingly soluble substrates is insufficient to provide the microorganisms with adequate substrate, resulting in substrate consumption and cell concentration limitations. However, a kinetic limited condition occurs in those bioreactors that provide sufficient mass transfer of substrates, but the cells do not balance their consumption with their transfer, resulting in the buildup of substrate to a saturation level, causing a substrate inhibitory effect [126]. Thus, another challenge of C1 gas bioconversion is the toxicity of the substrate CO as well as the product (solvent) to the biocatalyst. Increased productivities are obtained in bioreactors that offer a higher mass transfer rate and a higher achievable cell density. Enhancement of gas–liquid mass transfer certainly affects the applicability of the syngas fermentation process and strictly depends on the applied reactor configuration such as a stirred tank reactor, hollow fiber membrane reactor, bubble column, trickle bed reactor, gas–lift, bulk-gas-to-atomized-liquid contactor as well as the operational regimes such as semi-continuous, continuous gas feeding with fixed liquid volume or medium flow [127]. The main constraint on the overall rate of CO bioconversion is the very low solubility of CO and H_2_ in water at ambient temperature and pressure [39,128]. In this heterogeneous system, the primary barrier to mass transfer of the gaseous substrate from bulk gas to the reaction site is the liquid layer across the gas–liquid interface, and all other barriers are negligible [126].

Shen et al. (2014) [38] worked on the effectiveness of a monolithic biofilm reactor (MnBR) for syngas fermentation based on fluid flow patterns and CO mass transfers in abiotic conditions by using batch and continuous cultures with optimization of operational conditions such as syngas flow rates, liquid flow rates, and dilution rates. MnBR results showed a higher mass transfer efficiency and desirable biofilm development capacity compared to a conventional bubble column reactor (BCR). The novel MnBR design led to a higher volumetric mass transfer coefficient (*k_L_a*) than BCR. The syngas fermentation performance using *C. carboxidivorans* P7 in an MnBR system was evaluated based on the syngas utilization efficiency, ethanol concentration and productivity, and ratio of ethanol to acetic acid. It was remarked that the performance of the system was not only dependent on the mass transfer efficiency but also on the biofouling or abrading of the biofilm attached to the monolithic channel wall. Yasin et al. (2014) [129] investigated the effect of internal pressure and the gas–liquid interface area on the CO mass transfer coefficient using hollow fiber membranes (HFMBR) as a high mass transfer gas diffusing system for microbial syngas fermentation. The reported minimum value of *k_L_a* under abiotic conditions was the highest using submerged type HFMBRs, suggesting high potential as gas diffusing system for high gas–liquid mass transfer performance in syngas fermentation. Jang et al. (2018) [130] evaluated the bubble coalescence suppression driven carbon monoxide (CO)-water mass transfer increase by electrolyte addition in an HFMBR for microbial CO conversion to ethanol. They showed that the electrolytes that assisted mass transfer in HFMBR inhibited CO bubble coalescence, thereby enhancing the maximum *k_L_a* by a factor of 4.14. It was reported that the bioreactor operation using 2-(N-morpholino) ethanesulfonic acid-buffered basal medium (MBBM) with 1% MgSO_4_ led to a higher CO consumption, biomass, and ethanol production. Kinetic simulations also supported these findings. Sathish et al. (2019) [131] used a bulk-gas-to-atomized-liquid (BGAL) contactor combined with a packed bed to implement syngas fermentation. The authors mentioned that this application prevented the dispersion of gas-saturated droplets in the bulk liquid and found it to be energy efficient in transferring gas to the liquid phase, which enhanced the mass transfer for syngas fermentation.

De Medeiros et al. (2019) [132] applied a full dynamic modeling of syngas fermentation in a CSTR accounting for gas–liquid mass transfer and also substrate (CO, H_2_) uptake, biomass growth and death, acetic acid re-assimilation, and product selectivity. They remarked that the agitation rate also increased the mass transfer between the gas and the liquid allowing higher conversions and ethanol productivity. Devarapalli et al. (2017) [46] operated a trickle-bed reactor (TBR) in a trickle flow regime to create a very thin liquid film to come in contact with the gas phase. They used glass beads with a void fraction of 0.38, which was lower than the void fraction provided by other packing materials and reported that low void fraction decreases the availability of free space for gas–liquid mass transfer. A previous study by the same researchers also showed that TBR provided greater mass transfer capabilities compared to a CSTR [133]. Liu et al. (2019) [134] studied the gas–liquid mass transfer in a sparged and non-sparged CSTR with potential application in syngas fermentation and developed a model calculating *k_L_a* for syngas components CO, CO_2_ and H_2_, which could be used in selecting operating conditions in CSTRs. Almeida Benalcázar et al. (2020) [135] worked on a hybrid model for simulating ethanol production inside a 700 m^3^ bubble column bioreactor fed with gas of two compositions as pure CO and a 3:1 mixture of H_2_ and CO_2_. They reported a very strong dependency of process performance on mass transfer rates by using a model developed from oxygen transfer to water. In their work, *k_L_a* values were regarded for the possible presence of surfactants impeding the mass transfer; ethanol concentration, increasing the gas holdup; the ability of *C. ljungdahlii* and *C. carboxydivorans* forming biofilms and enhancing mass transfer via the circulation of bacteria inside the bioreactor. When the H_2_/CO_2_ mixture was fed to the bioreactor, they found 19% lower productivity of CO fermentation, gas utilization up to 23% and 17% for H_2_/CO_2_ and CO fermentations, respectively, and ethanol productivity up to 5.1 g/L.h. However, at the same process conditions with H_2_/CO_2_ mixture feeding, they obtained ethanol productivity and gas utilization up to 9.4 g/L.h and 38% in the case of mass transfer coefficients that were 100% higher than those estimated.

Although the boosting of gas–liquid mass transfer varies depending on the applied reactor configuration as mentioned above, some studies showed that the addition of activated carbon and nanoparticles in syngas fermentation medium stimulated gas solubility as well as enhanced product formation. Atiyeh et al. (2016) [136] reported the activated carbon addition to a CSTR led to higher ethanol production of 19 g/L compared to the control reactor operated in a medium without active carbon addition, which was about 1 g/L. Kim and Lee (2016) [137] exhibited the positive effect of nanoparticle addition on the gas solubility and ethanol production. They used magnetic silica nanoparticles with Co and Fe oxides to increase CO, H_2_ and CO_2_ solubility, and cell mass, alcohol and acid production during syngas fermentation. Furthermore, they reported that CO, H_2_ and CO_2_ solubility were about 315%, 294%, and 97%, respectively, while the production of ethanol, acetic acid and cell mass were 214%, 60% and 228%, respectively, compared to the control reactor.

## 9. Biofilm Formation

To understand and predict the microorganisms’ behavior in gas fermentations, most of the mathematical models have focused on biofilm formation related to the microbial metabolism [135]. As a well-known process, bioreactor performance is affected by biomass retention. As higher retention times can lead to clogging problems, granulated shapes of biofilms were recommended in air-lift bioreactors [135,138]. Although planktonic growth has been documented mostly in syngas fermentation, the capacity of *C. ljungdahlii* to produce biofilms under stress caused by NaCl addition has also been observed as a biological reaction to the stress [139]. Ebrahimi et al. (2005) [140] pointed out that a potential problem of monolith reactors was clogging due to biofilm formation. They investigated the formation and removal of biofilms in a monolith reactor consisting of ceramic material and showed that the formation might be minimized by using appropriate operating conditions. Sathish et al. (2019) [131] used a BGAL contactor combined with a packed bed, which was randomly packed with polypropylene BioTube packed material in syngas fermentation. They found that by immobilizing the syngas fermenting culture in the packed bed below the liquid dispersing zone, the biofilm directly consumed the substrates from the liquid flowing through the packed bed. Devarapalli et al. (2017) [46] used a trickle-bed reactor (TBR) for ethanol production with continuous syngas fermentation. The reactor had 6-mm soda lime glass beads as packing material with a void fraction of 0.38. This fraction was reported as lower than those provided by other packing materials such as intalox saddles (0.6 to 0.9) and pall rings (0.9). They concluded that the low void fractions led to reductions in free space for gas–liquid mass transfer and the reactive holdup volume and mentioned that different cell immobilization techniques for the packing materials could enhance the biofilm formation in short times. The packing materials affect the gas–liquid mass transfer as well as the applied reactor configuration.

Ammam et al. (2016) [141] investigated the biosynthesis of ethanol by *S. ovata* and the enhancement of the acetate production rate by optimizing trace elements in the cultivation medium. They worked on acetate and ethanol productions from CO_2_ with H_2_ or a cathode as the electron source with different concentrations of trace elements in the medium. In the microbial electrosynthesis (MES) setup, they used H type reactors and the graphite sticks as cathodes. The conversion of CO_2_ to acetate by MES was greatly stimulated by the optimization of tungstate concentration in *S. ovata* cultivation medium in addition to the production of ethanol during autotrophic growth on H_2_:CO_2_ or by MES.

Batlle-Vilanova et al. (2017) [30] studied MES of butyrate from carbon dioxide by using a two-chambered tubular reactor equipped with a commercial carbon cloth as a cathode. They showed the production of butyrate as the primary organic end product of MES from CO_2_. Haas et al. (2018) [142] developed a system including solar-powered electrochemical reduction of CO_2_ and H_2_O to syngas, followed by fermentation, by using a commercially available silver-based gas diffusion electrode as the cathode in the CO_2_ electrolyzer. The out-coming syngas from the electrolyzer was converted to desired alcohols such as butanol and hexanol with high carbon selectivity. In their work, the conversion of photovoltaic electricity, CO_2_ and H_2_O to the alcohols was reported up to 100% Faradaic efficiency. These studies show that these systems as hybrid applications are promising for further research on the production of different products via MES and syngas fermentation.

## 10. Kinetics

For bioprocesses to be efficient and yields to be increased, a thorough knowledge of the underlying kinetics of the biocatalyst is required. When kinetic information is used to process a model for optimizing the reactor design and bioprocesses, product yields may be substantially increased. Validating kinetic design parameters requires a systematic approach using semi-continuous or batch reactors. Conducting a kinetic analysis on purely anaerobic or axenic cultures is often time-consuming and labor-intensive and may sometimes cause complications during scale-up operations. Gaseous substrates will also provide some challenges due to mass transfer restrictions [143].

The Haldane kinetic model, a modified version of the Monod equation, is used to estimate kinetic parameters such as the half-saturation constant for CO ($K_{S}$) and substrate inhibition constant ($K_{I}$) of CO fermentation [144].

$$\mu ={\;\mu }_{\max}\left(\frac{\mathrm{CO}}{\mathrm{CO}+{\;K}_{S}+\frac{{\mathrm{CO}}_{2}}{K_{I}}}\right)$$

where μ is the specific cell growth rate (h^−1^), $\mu_{\max}$ is the maximum specific cell growth rate (h^−1^) and CO is the liquid concentration of CO under equilibrium conditions with the gas phase (mg/L).

The optimal CO partial pressure for *C. carboxidivorans* growth with no pH control was reported to be 1.1 atm, which corresponds to a dissolved concentration of 25 mg/L for a liquid to gas volume ratio (V_L_/V_G_) of 0.28 and 0.92 used for the study [144]. Given the fact that the metalloenzymes involved in the WL pathway could be inhibited by a higher dissolved CO concentration in the liquid phase, this must be considered while designing and operating a syngas fermentation bioreactor. Another component of syngas is CO_2_, which is also a possible inhibitor for fermentation due to its ability to lower the medium’s pH through the production of carbonic acid.

Mohammadi et al. (2014) used an additive model by combining the Luong kinetic model for CO and Monod for H_2_ to describe the growth of the *C. ljungdahlii* in batch bottles pressurized with syngas. The Andrews model [145] and modified Gompertz equation [145,146] were used to describe the CO uptake rate and product formation (ethanol and acetate). The qmax, the maximum CO uptake rate obtained in the study, was 34.364 mmol/g_cell_/h [145].

Candry et al. (2018) [64] developed a high-throughput anaerobic growth curve method in combination with a data analysis technique for estimating the growth rate and kinetic parameters across a range of substrate and product concentrations for *C. kluyveri.* The maximum growth rate (µmax) was found as 0.24/h, with a half saturation index, 3.8 mM, for acetic acid and the inhibitory concentration of butyrate was found as 124.7 ± 5.7 mM. A hexanoic acid toxicity concentration of 91.3 ± 10.8 mM at pH 7 was determined. The product profiles were analyzed using a 96-well plate vs. Balch tubes.

It is critical to ascertain the kinetic properties of hydrogenotrophic methanation systems in order to understand the material flux from gaseous substrate to methane. However, the hydrogenotrophic activity analysis is still uncommon and unstandardized [147]. Ripoll et al. (2020) [147] studied an assay design for hydrogenotrophic activity with the full calculation based on the kinetics of H_2_/CO_2_ conversion to methane. The equation below was suggested to calculate inoculum size, which can also be applied to various types of biological sludges from wastewater plants to solid digesters

$$V_{B}=\frac{-∆PV_{hs}C\;}{4RT∆tXk_{m}}$$

where $V_{B}=$ is inoculum size (L), ∆i (atm) is pressure depletion inside the headspace, $V_{hs}$ is the headspace volume (L), C is 64 g_COD_/mol, which is the conversion factor from moles of methane to g COD, *R* is the universal gas constant (L atm/molK), *T* is temperature (K), ∆t is time (days), *X* is biomass concentration (g_VSS_/L) and $k_{m}$ is maximum specific rate for substrate consumption (g_COD_ g_VSS_^−S^ d^−d^).

Another research was conducted to determine the kinetics of continuous methane generation utilizing CO_2_ and H_2_ in mixed cultures. CSTR reactors were run at various H_2_ loading rates (2–14 m^3^ H_2_/m^3^/d) and hydraulic retention times (HRT) ranging from 5 to 30 d. The composition of the feeding gas (H_2_:CO_2_) was maintained at 80/20. A kinetic study using the Monod equation revealed that hydrogenotrophic methanation cultures had a specific growth rate of 0.18/d [148].

## 11. Electron Transfer Mechanism in Bioelectrochemical System

In the BES literature, it was suggested that the electrons can flow from the bacteria to electrodes (electrogen) or from electrodes to bacteria (electrotrophs), namely in bacteria from the genus *Shewanella* and *Geobacter* [88,149]. In BES for the production of valuable compounds (bioelectrochemical synthesis), the electrons flow from electron-donor electrodes to microorganisms. This process is called cathodic extracellular electron transfer (EET) [150]. The essence of this mechanism is similar to the anodic EET, but the knowledge about this is far from being fully understood. The electron interaction between the electrode and the microorganism can occur as DET or involve dissolved species in mediated electron transfer (MET) [27].

DET implies the direct contact between the electrode and bacteria by nanowires and/or membrane bound redox proteins [151]. Filamentous conductive pili are involved in electron transfer in *Shewanella* [152] and *Geobacter* [153]. DET is also reported in another microbial community from beta proteobacteria and firmicutes [154]. Multiheme c-type cytochromes, namely OmcA (involved in the inner membrane), CymA (a link point between the inner membrane and the periplasm), MrtA (present in the periplasmic) and MtrC (located on an extracellular site of the outer membrane) were described as crucial components in DET in gram-negative bacteria [149,155].

In MET, a mediator accepts the electrons from the electrode and transfers these to electrodes. The redox mediators can be exogenous and excreted by bacteria or artificial. The exogenous mediators can be secondary metabolites shuttled via the outer cell membrane cytochromes and via periplasmatic/cytoplasmatic redox couples, or primary metabolites via reduced terminal electron acceptors (anaerobic respiration) and oxidation of reduced fermentation products [153]. Rubredoxin, hydrogenase, formate dehydrogenase [156] and membrane-bound NADH: ferredoxin oxidoreductase [157] are released from cells and adsorbed onto electrodes to accept electrons. Artificial mediators as methyl viologen [158], anthraquinone-2,6-disulfonate [159], or neutral red [160] are largely used in bioelectrochemical synthesis.

## 12. Industrialization and Patents

### 12.1. Syngas Fermentation

Fermentation of insoluble gaseous substrates (CO and H_2_) is challenging because these substrates must be dissolved in the media before the microbe can use them. Numerous methods and equipment have been investigated in order to increase the volumetric mass transfer coefficient. A fermentation process using CSTR with at least one gas dispersion impeller (Rushton impeller or concave impeller) and one mixing impeller (marine impeller or marine propeller) has been reported to offer effective mass transfer when maintaining a pressure of at least 1 psig with syngas. The reactor vessel’s boot, which contains a vortex breaker, assists in preventing gas from being pulled out via the medium outlet (US9976158B2). A multiple-pass trickle bed (MP-TBR) configuration enables the treatment of nitrogen-rich producer gas or waste gas without the need for a pressurized reactor vessel or a larger reactor vessel by increasing the recirculation and turbulence of the gaseous substrate via sections equipped with a gas circulation fan and packing media (US 20210079326A1, WO2019046188A1).

LanzaTech Inc. (Skokie, IL, USA) is the global leader in commercializing syngas fermentation technology. The company successfully launched in May 2018 its first commercial bioethanol production plant in China in collaboration with the Shougang Group (Beijing, China), using waste gas from the Jingtang Steel Mill (Hebei, China). The plant has the capacity to produce 46,000 metric tons of bioethanol per year. Lanzatech have signed agreements to enter a partnership with ArcelorMittal (Ghent, Belgium), Swayana (Pretoria, South Africa) and Indian Oil Corp. Ltd. (Haryana, India) for commercialization of their gas fermentation technology. The company’s proprietary microorganisms and technologies are used to recycle waste gas from sectors such as steel manufacturing, and other wastes into CarbonSmart™ products (ethanol and other commodity chemicals). The detailed information from 2005, when it was founded, to 2017 can be read elsewhere in the case study published by Karlson et al. 2018 [161]. Once completed in 2022, the “Steelanol project” is expected to produce 80 million litres of ethanol using waste gas from the ArcelorMittal steel plant in Gent, Belgium. The steelanol plant is powered by Lanzatech fermentation technology, Primetals Technologies (Linz, Austria) engineering, and E4Tech (London, UK) life-cycle assessment [162]. LanzaTech has achieved many milestones in recent years in collaboration with several industry partners. LanzaTech has cooperated with India Glycols Limited (Uttar Pradesh, India), Far Eastern New Century (Taipei, Taiwan), and Lululemon Athletica (Vancouver, Canada) to create the world’s first fabric made entirely of polyester derived from carbon emissions [163]. LanzaTech and a chemical company, BASF (Ludwigshafen, Germany), collaborated to synthesize n-octanol on a laboratory scale from CO and H_2_ [164]. In partnership with Unilever (London, UK) and India Glycols Limited (Uttar Pradesh, India, LanzaTech is set to bring into the market the first laundry capsule using the surfactants made from carbon emissions by 2030 [165]. In addition, Coty Inc. (New York, NY, USA) will collaborate with LanzaTech to use sustainable ethanol in their major fragrance production by 2023 [166]. By 2024, a collaboration between LanzaTech, TotalEnergies (Courbevoie, France), and L’Oréal (Clichy, France) aims to utilize shampoo and conditioner bottles manufactured from recycled carbon, according to the company [167].

LanzaJet (IL, USA) is a LanzaTech’s spin-off company that produces sustainable aviation fuel (SAF) and renewable diesel in cooperation with Mitsui & Co. (Tokyo, Japan), Suncor Energy Inc. (Calgary, Canada), and All Nippon Airways (Tokyo, Japan), was launched in June 2020. The LanzaJet alcohol-to-jet (ATJ) technology uses any waste as a source of ethanol, including municipal solid waste (MSW), agricultural residues, industrial off-gases, and biomass, and then converts it to Synthetic Paraffinic Kerosene (SPK) and Synthetic Paraffinic Diesel (SPD) via dehydration, oligomerization, hydrogenation, and fractionation [168]. LanzaTech successfully demonstrated the use of sustainably generated ethanol from industrial waste gas in October 2018 by mixing it with jet fuel to power a Virgin Atlantic aircraft from Orlando to Gatwick [169].

### 12.2. Microbial Chain Elongation

Since the past decade, bioprocesses for chain elongation have advanced significantly [12]. Apart from being an efficient source of energy, additionally, medium chain fatty acids (MCFAs) may be utilized as animal nutrition additives, chemical additives for plasticizers and coatings, and agrochemicals for crop preservation. There are pilot-scale and industrial-scale manufacturing facilities for medium or short chain fatty acids [170]. Numerous different pathways for chain elongation have been proposed, primarily using waste materials as a starting point. However, with the growing interest in carbon capture and utilization, industrialization processes are increasingly focused on the production of C1 and C2 hydrocarbons from waste gas and subsequent chain elongation, avoiding the distillation step required for ethanol recovery. Wageningen University (Wageningen, The Netherlands) patented the enzymatic synthesis of C6-C18 fatty alcohol and C8-C18 fatty acids utilizing gas substrates in 2007 (EP2271764B1, US8431368B2). INVISTA North America S.A.R.L. (Delaware, USA) has granted a patent on the synthesis of 7-C compounds through C1 carbon chain elongation in association with coenzyme B synthesis (US9580731B2). A recent patent on methods of producing caprylic acid and/or caprylate by Cornell University (Ithaca, NY, USA) using chain-elongating bacteria from ethanol and gas substrates was published. High productivities were achieved by changing the ratios of ethanol and acetate, extracting caprylate products and acclimatizing the chain elongating bacteria (US10526624B2). 

The CAPRA project aimed to upgrade the syngas fermentation effluent to MCCAs, bringing forward the biological chain elongation technology from lab to pilot scale. The project brings together academic and industrial partners [VITO (Mol, Belgium), ArcelorMittal (Ghent, Belgium), OWS nv (Ghent, Belgium), Proviron nv (Hemiksem, Belgium) and Ghent University (Ghent, Belgium)] to join their efforts on developing a process chain starting from using greenhouse gases to produce caproic acid [171]. ChainCraft, based in the Amsterdam, Netherlands, focuses on the biosynthesis of long chain acids for a variety of applications through chain elongation techniques [172].

### 12.3. Hydrogenotrophic Methanation

Hydrogenotrophic methanation is another energy-intensive process for converting C1 gases to valuable biofuels. Patents for this method stretch all the way back to 2007. Several distinct processes for the conversion of C1 gases to methane have been patented. This process is also known as biological methane upgrading since it makes use of hydrogen methanation bacteria. A patented technique for converting CO_2_ to CH_4_ via the use of methanogenic archaea can be found elsewhere (EP2032709B1). Hydrogenotrophic methanogenesis of H_2_ and CO_2_ into CH_4_ was granted patent as a system saving 10% natural gas using methanogenic microorganisms by Rohöl-Aufsuchungs Aktıengesellschaft (Vienna, Austria) in 2019 (EP3280807B1). A patent from the University of Seoul (Seoul, South Korea) described a reactor system in which hydrogen methane bacteria and organic acid methane bacteria coexist as dominating species for hydrogen methanization proliferation. This innovation enables low-pressure operation of a hydrogen methanization bacterium incubator, thus improving its economic efficiency and safety (KR102059924B1). The University of Denmark (Lyngby, Denmark) and Vestforsyning A/S (Holstebro, Demark) have filed patent on a hydrogen-based biogas upgrading system that uses anaerobic reactors to convert CO_2_ and H_2_ to CH_4_. Acidic waste was used as co-substrate, and in the end, CO_2_ content in the reactor was reduced during biogas production (CN103958688A, US20140342426A1). Hydrogenotrophic methanation may be industrialized by using waste gases from large-scale industries. For instance, Electorcheae (Planegg, Germany) has filed a patent on the use of industrial CO_2_ containing gas for methane enhanced gas production in 2018 (WO2020089181A1). Suez Groupe (La Defense, France) has filed a patent on a syngas biomethanation apparatus and method in 2017 under the number EP3418371A1. Three ES S.r.l. (Lazzate, MB, Italy) used hydrodynamic cavitation for biological methanation of gaseous substrates. A biological methanation plant is described in this patent application including three steps: providing biomass, supplying H_2_ and CO_2_, and lastly dissolving the biomass through hydrodynamic cavitation and utilizing that biomass to convert H_2_ and CO_2_ to methane (EP3613708A1).

### 12.4. Bioelectrochemical Synthesis

Bioelectrochemical synthesis could be a more viable application of BES. The potential of continuous CO_2_ emissions in combination with several environmental concerns created a big move towards the development of novel technologies for CO_2_ capture and higher value organic molecule generation [173]. Bioelectrosynthesis of methane [24], acetate [29], ethanol [174], propionate [175] and butyrate [176] are examples of products electrosynthetised by microorganisms from CO_2_. H_2_ generation from wastewater [177] has attracted the attention of bioelectrosynthesis researchers. The transition from laboratory to industrial scale has been gradual, and some demands have an effect on overall performance, including resistance, electrode spacing, membrane location, and overpotentials [178].

A 10 L pilot-scale hydrogen bioelectrosynthesis system using domestic wastewater was tested. Two independent MEC cells in series were operated at ambient temperature [179]. Carbon felt was used as the anode (10 × 5 cm) (SGL Group, Kitchener, ON, Canada) and Sigracet GDL 25 BC Carbon paper (SGL Group, Kitchener, ON, Canada) with electro-deposited Ni particles (0.25–0.30 mg-Ni/cm^2^) as the cathode. Polyester cloth was used to separate the electrodes. A total of 2.6 L/L/day of H_2_ was obtained with 23% of Coulombic efficiency.

A 100 L MEC with six separate cell cassettes that work individually and in parallel was assessed with raw domestic wastewater [180]. Each cassette had two carbon felt anodes (0.2 × 0.3 m) with 10 mm thickness (Olmec Advanced materials Ltd., Sheffield, UK) connected to a stainless-steel mesh as the current collector. The cathode was stainless steel wool (Merlin Ltd., Wiltshire, UK). The wool was wound with stainless steel wire. The ratio anode/cathode was 5:1. The physical separation between them was by a Rhinohide membrane (Entec Ltd., Harrogate, UK). The reactor was operated for 12 months at ambient temperature and produced an average of 0.6 L/day of hydrogen; however, energy recovery (48.7%) and Coulombic efficiency (41.2%) was around half that needed for energy neutrality [180].

A 1000 L continuous flow MEC with 24 electrode modules in series was developed for winery wastewater treatment [181]. Each electrode module contains six anodes and six cathodes. Anodes were made of graphite fiber brushes with titanium wire core (5.1 × 66 cm) (Gordon Brush, CA, USA). Two solid strips of SS 316 were used as current collectors. Strips of glass fiber (Nippon Sheet Glass Co, Ltd., Tokyo, Japan) were used to separate the anodes and avoid closed circuits. Cathodes were made of SS 304 mesh (7.6 × 66 cm) (McMaster-Carr, OH, USA). Gas production reached a maximum of 0.19 ± 0.04 L/L/day at 31 ± 1 °C, although most of the product gas was converted to methane (86 ± 6%) [181].

The methane production via bioelectromethanogenesis in a 50 L reactor was 0.23 mmol/l/d with a pure culture of electroactive methanogens, *Methanococcus maripaludis* [182], 1/6 of that observed in the lab scale reactor. Twenty modules, in a circular configuration, comprised the pilot scale with carbon laying electrodes (HP textiles, Schape, Germany) as working electrodes in the inner chambers, and a counter chamber with 20 sheets of carbon fabric electrodes, which are placed circularly around the working chamber. FKS-PET-130 cation exchange membranes (FUMATECH BWT GmbH, Bietigheim-Bissingen, Baden-Württemberg, Germany) were used between the chambers. The gas flux of pure CO_2_ was set to 1.5 L/min. The energy efficiency was 27% [182]. Siemens and Evonik (Essen, Germany) are now building a test plant at the Evonik facility in Marl, Germany, to electroreduce CO_2_ to CO and posteriorly to butanol and hexanol [183].

## 13. Techno-Economic Analysis and Life Cycle Analysis

Bioethanol production from renewable sources of feedstock such as sugar, starch, or lignocellulosic materials creates some drawbacks on the feasibility of technological applications due to the high value of these crops as a food product. However, the utilization of inexpensive feedstock such as municipal solid wastes, green waste, and agroindustry wastes for bioethanol production can reduce the costs of these applications in addition to lessening the dependency on the fossil fuels [39]. Considering the tremendous increase in global ethanol production from 46.5 to 102.8 Mm^3^ between the years of 2007 and 2019 (RFA, 2020), the bioethanol production from inexpensive non-food feedstocks has become more important than the production from food feedstocks such as corn and sugar cane. In addition to this, the marketing price of ethanol from inexpensive feedstocks should be at least as good as the corn or sugar cane ethanol price [127]. Therefore, the process economy is very critical. Phillips et al. (2017) highlighted that its economy, in the case of ethanol production via the fermentation process from syngas, is strictly related to the improvements in energy efficiency in terms of retaining the higher heating value from the gasification products, increasing the fermentation product yield, and the use of energy efficient separation technologies such as membrane separation [128].

De Luna et al. (2019) [184] reported that electrochemical carbon dioxide reduction (CO_2_R) has been gaining significant attention as another sustainable pathway for producing fuel and chemical feedstocks. The authors showed the Faradaic and energy conversion efficiencies for many CO_2_R products and presented the techno-economic analysis results of hydrogen, carbon monoxide, ethanol, and ethylene costs as a function of electrolyzer energy conversion efficiency and electricity costs. The main finding from this evaluation was about the extreme variability of the chemical prices regarding the geographic region and feedstock used. Among the different pathways for converting CO_2_ to chemicals such as C1 or multi-carbon (C2+) oxygenates, and hydrocarbons, direct synthesis of higher alcohols from syngas is considered as a superior approach from environmental and economic standpoints. For industrial applications, the usage of different catalysts such as electrocatalysts can stimulate the process efficiencies to produce C1 to C3 molecules and H_2_. However, there are different factors affecting the economics of electrocatalytic processes, including the availability and price of renewable electricity, the regional cost of feedstock and traditional petrochemical manufacture, and economic incentives to transition to low-carbon processes [184].

As is well-known, CO is the byproduct of many thermochemical, biological, and electrochemical processes and can be evaluated via different beneficial usage alternatives. For example, CO alone or as a mixture with H_2_ in the syngas can be used as feedstock for Fischer–Tropsch synthesis and the fermentation cycle; electrochemical CO_2_R or sequential pathways such as syngas electrosynthesis and biocatalysis. The applicability of each method can be determined by the elucidated techno-economic analysis, including process description, proper assumptions, and the selection of modeling parameters. Many techno-economic analyses have been performed for each pathway with different alternative processes by using simulation software since this analysis includes different components and produces different information such as energy efficiency, power demand, capital and operating cost. Sun et al. (2019) [127] indicated that these software programs (e.g., Aspen Plus) are capable of producing performance evaluations on the product cost and prices, in addition to environmental impact assessment and/or life cycle assessment of the processes. Perales et al. (2011) [185] worked on the techno-economic analysis related to thermochemical conversion of biomass to ethanol for two gasification technologies as circulating fluidized bed gasification and entrained flow gasification. They developed different scenarios and classified the current scenarios as available technologies and state of-the-art mixed alcohol catalysts (RheMn/SiO_2_ and KCoMoS_2_ catalysts) while future scenarios followed the effects of improvements in MoS_2_ catalyst performance and availability of pressurized solid biomass feeding systems. Aspen Plus 2006.5 was used as a simulation tool for solving material and energy balances, and the production cost of ethanol from lignocellulosic biomass was determined based on the simulation results from Aspen Plus. They showed that the minimum ethanol selling price (including 10% rate of return) was about 0.90–1.25 $/L ($=US Dollar) for current catalysts while it was found as 0.71 $/L for enhanced MoS_2_ catalyst performance in a future scenario. It was also remarked that the minimum ethanol selling price would decrease to 0.55 $/L if biomass piston feeders were commercially available.

Since previous studies ([184,186] showed that the costs of ethanol production are lower than <EUR 1000/ton by applying current industrial processes, the newer alternative pathways for ethanol production need more research in terms of process stability and economical application. Hossain et al. (2019) [187] have reported the ethanol production costs for biochemical and thermochemical routes as $ 164.4 million and $ 151.9 million for the annual processing of 0.658 million tons of corn stover, respectively. However, the thermochemical pathway led to an additional 64.8 million liters of ethanol production. De Luna et al. (2019) [184] reviewed the techno-economic analysis of various alcohols regarding their processing costs for different pathways and indicated that the processing cost of ethanol for electrocatalytic and biocatalytic processes are $ 515 and $ 670 per ton, respectively. The ethanol prices for second-generation biochemical ethanol via enzymatic hydrolysis, syngas fermentation, direct and indirect thermochemical were reported as $ 0.95/L, $ 0.30/L, $ 0.71/L, $ 0.56/L [185,188,189,190].

Research studies related to new technological developments should be supported by techno-economic evaluations considering the market penetration difficulties for bioethanol production from non-food feedstocks. However, there are very limited studies on these evaluations. Beyond the techno-economic analysis, life cycle assessment (LCA) of these processes has become more critical for environmental sustainability, especially the latest issues on climate change effects related to the greenhouse gas emissions. Müller et al. (2020) [191] indicated the proper estimation of the carbon footprint of CO_2_ by LCA, which is standardized according to ISO 14040, 14044, and 14067. The Global CO_2_ Initiative and the U.S. Department of Energy, National Energy Technology Laboratory modified these standards for CO_2_ utilization in LCA guidelines. They have also been linked to techno-economic analysis capable of holistic assessment of LCA and economic considerations for CO_2_ utilization [191]. Various syngas utilization routes should be assessed based on the multiple environmental impacts such as ozone depletion, eutrophication, global warming, etc. Previous studies showed that biocatalytic syngas fermentation led to the production of more valuable chemicals, including ethanol, butanol, and biodegradable polymers such as polyhydroxyalkanoates (PHAs) with low CO_2_ emissions ranging from 0.26 to 0.45 tonnes CO_2_/tonne product and the cost of PHA production was $ 1650/tonne. However, FT synthesis having higher rates of production with low production costs caused higher CO_2_ emissions 3.8 tonnes CO_2_/tonne product, resulting in diesel costs of $ 240 to 525/tonne [184,192,193,194,195,196].

Sternberg et al. (2017) [197] made LCA of CO_2_-based C1-chemicals formic acid, carbon monoxide, methanol, and methane based on the reduction of global warming and fossil depletion impacts using 1 kg of H_2_. For example, the authors evaluated several hydrogen supply processes relating the maximum environmental impact reductions to existing and proposed hydrogen supply processes via steam-methane-reforming (SMR), thermal processing, and water electrolysis. The global warming impacts of the hydrogen supply by these processes were accounted for as 10.6 kg CO_2_-eq per kg H_2_, 7.9 kg CO_2_-eq per kg H_2_, and 0.4 kg CO_2_-eq (wind electricity) to 18.5 kg CO_2_-eq (grid mix EU-27 in 2020). They found that CO_2_-based production of formic acid had the highest environmental impact reductions, followed by carbon monoxide and methanol, while the lowest ones were obtained for CO_2_-based methane production. However, the authors indicated that the environmental impacts of CO_2_-based production of formic acid could be diminished even if hydrogen was supplied by fossil-based SMR.

De Luna et al. (2019) [184] presented an LCA for electrochemical synthesis of common carbon-based commodity chemicals such as formic acid, carbon monoxide, ethanol, and ethylene. The authors mentioned that ethylene had the largest global market size of around EUR 230 billion corresponding to the highest emission production as 862 Mt CO_2_-eq per year. The authors indicated that the electricity grid carbon intensity as CO_2_ per kWh of electricity generated and the energy conversion efficiency were the most sensitive factors affecting overall CO_2_ emissions. In the case of neglecting the capital costs for the process, such as construction and electrode materials, the authors found that carbon monoxide and formic acid resulted in carbon emissions lower than fossil fuel-derived sources. In a comparison of electrocatalytic, biocatalytic, and traditional fossil fuel-derived processes for ethylene, carbon monoxide, ethanol, and formic acid production, the carbon emissions as tonne CO_2_-eq/tonne produced were found as higher values in the electrocatalytic process than those in the biocatalytic process, which were even negative values. In their work, it was highlighted that the electrosynthesis was competitive with fossil fuel-derived feedstocks and the electrical-to-chemical conversion efficiencies and electricity costs should be at least 60% and lower than 4 cents/kWh, respectively [184]. To determine the technical challenges and economic barriers for scaling up of the syngas fermentation operation, techno-economic analysis is very important. It should be considered together with LCA since the fermentation technology is promising for the production of biofuels and value-added chemicals from different feedstocks with a neutral or negative carbon footprint to support the fuel, energy, chemical, agricultural and environmental industries [127].

## 14. Conclusions

This study focused on gathering information on different reactors used for the conversion of microbial C1 gas through conventional and bio-electrochemical routes. Recently, a transition from CSTR to attached growth bioreactors such as membrane and trickling bed has been observed. Forming a thin layer of liquid enables the C1 molecules to overcome their mass transfer limitation to the microorganisms. Apparently, by using attachment growth, a high concentration of microbial biomass may be maintained inside the system, consequently increasing the process’s productivity. The production of valuable chemicals from CO_2_ and electricity through electroactive microorganisms in bioelectrochemical cells has received considerable interest recently as a sustainable method of turning surplus energy produced from renewable energy sources into stable commodities. Internal resistance, membrane fouling, and pH variations are all obstacles that must be addressed. From laboratory to industrial scale applications, a scalable electrode and reactor design must be developed.

## Figures and Tables

**Table 1:** Merits and drawbacks of different bioreactor configurations used for C1 gas fermentation.

Bioreactor Type	Process			Merits	Drawbacks
	Syngas Fermentation	Chain Elongation	Hydrogenotrophic Methanation		
Continuous Stirred Tank Reactors (CSTR) [31,67,68,69,71]	✓	✓	✓	Flexible for many bioprocesses Control on gas–liquid mass transfer	Commercialization is not cost effective Scale up increases energy requirements
Biofilm Formation Reactors [32,33,34,70,72,73,74]	✓	✓	✓	High biomass concentration Smaller reactor volumes Low energy requirements	Limitation on mass transfer with increasing biomass concentrations
Rotating Packed Bed Biofilm Reactors [35,43,44]	✓			Efficient mass transfer from bulk gas to cell surface	The rate-limiting step is the diffusion across gas–liquid interface Maintaining optimum rotation needs careful operation
Monolithic Biofilm Reactor [36,37,38]	✓			Prevents biomass wash out at greater dilution rates Large pore size Specific surface area Great mechanical strength	Dependence on channel geometry Low flow rate of gas
Membrane Bioreactor [40,41,77,78]	✓		✓	Suitable for poorly water soluble gases Flexible application	Membrane wetting and biofouling
Trickle Bed Reactor [45,46,47]	✓			Large volume/surface area No need for mechanical agitation Control on superficial gas velocity	Inconsistent irrigation of packing material
Bubble Column Reactor [49,50,51,52,53]	✓			Low maintenance and operational costs No need for mechanical mixing Operation in different modes	Optimization of bubble size for a successful mass transfer
Hollow Fiber Reactors [42,65]	✓	✓		Improved production rates Lower investment costs Resistance to washout of microorganisms	Uncontrolled thickness of biomass can limit mass transfer
Carrier Bed Reactors [75,76,77,78,79]			✓	Different types of carriers can be used such as biochar, polyurethane foam, etc.	Need for mechanical agitation
Fixed Bed Reactors [76]			✓	Low operation costs Low reactor size Improved biomass concentrations	Gas–liquid mass transfer limitations Channeling

## Data Availability

Not applicable.
